# Central Abnormalities in Idiopathic Tinnitus With Mild Hearing Loss: Selective Attention and Preprocessing Dysfunction

**DOI:** 10.1002/brb3.71308

**Published:** 2026-03-12

**Authors:** Xingqian Shen, Hui Pan, Yingzhao Liu, Ziying Xu, Xiaoye Chen, Qin Liu, Kaijun Xia, Wen Xie, Linlin Wang, Bo Liu, Hongjun Xiao

**Affiliations:** ^1^ Department of Otorhinolaryngology‐Head Neck Surgery and ENT Institute, Union Hospital, Tongji Medical College Huazhong University of Science and Technology Wuhan China; ^2^ Hubei Province Clinical Research Center for Deafness and Vertigo Wuhan China

**Keywords:** auditory event‐related potentials, idiopathic tinnitus, mild hearing loss, mismatch negativity, P300

## Abstract

**Objectives:**

To verify the abnormal brain electrical activity of idiopathic tinnitus through auditory event‐related potentials and elucidate the impact of mild hearing loss.

**Design:**

Auditory event‐related potentials were collected from 74 patients with idiopathic tinnitus as the primary complaint (divided into a normal hearing group of 39 and a mild hearing loss group of 35 based on hearing level) and 37 healthy volunteers. Various auditory event‐related potential indicators were compared among the groups, and the correlation between these indicators and the clinical characteristics of idiopathic tinnitus patients was analyzed.

**Results:**

Idiopathic tinnitus patients with normal hearing have increased P1 amplitude, delayed P300 latency, decreased P300 amplitude, and decreased mismatch negativity (MMN) amplitude. Idiopathic tinnitus patients with mild hearing loss have further reduced P300 amplitude and delayed MMN latency. In addition, the P300 latency of idiopathic tinnitus patients is significantly correlated with the duration of tinnitus and Tinnitus Handicap Inventory (THI) score, the P300 amplitude is significantly correlated with the Pittsburgh Sleep Quality Index (PSQI) score, and the MMN latency is significantly correlated with the duration of tinnitus.

**Conclusion:**

Patients with idiopathic tinnitus exhibit abnormalities in multiple auditory event‐related potentials, with mild hearing loss exacerbating some of these abnormalities. Idiopathic tinnitus and mild hearing loss may lead to selective attention and preprocessing dysfunction.

## Graphical abstract

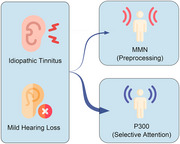

## 1 Introduction

Tinnitus refers to a subjective perception of meaningless sounds perceived by patients themselves in the absence of an external sound source (De Ridder et al. 2021). Most tinnitus sound perception is subjective and cannot be located at the physical sound source within the patient's body. After excluding a series of related diseases that may cause tinnitus, such as earwax embolism, otitis media, acoustic neuroma, and systemic diseases, this type of unexplained tinnitus is referred to as idiopathic tinnitus (Tunkel et al. 2014). Increasingly, more scholars believe that the lesions of idiopathic tinnitus are not limited to the ears, and plasticity changes in the central nervous system may play a crucial role in the pathophysiological mechanism of idiopathic tinnitus (Shore et al. 2016). Among these, the neurophysiological abnormalities associated with tinnitus have received extensive attention. For example, studies have found that the increase in spontaneous firing rate of the brain neurons and changes in neural synchrony are closely related to tinnitus‐like behavior in animals (Baguley et al. 2013).

Event‐related potentials (ERPs) are assigned special psychological meanings to certain stimuli and use multiple or diverse stimuli to induce cranial potentials, reflecting the neurophysiological changes in the brain during cognitive processes. ERPs are brain potentials recorded from the surface of the head when people process a certain stimulus cognitively. Auditory event‐related potentials (AERPs) are the use of sound stimuli to induce changes in these potentials, in order to study the neurophysiological activity of the brain during auditory cognition. The AERPs belong to the cortical auditory evoked potential (CAEP), which records exogenous components influenced by the physical properties of sound stimuli and endogenous components reflecting auditory cognitive processing such as discrimination, cognition, and decision‐making. Exogenous components include P1, N1, and P2, which reflect early neural activity during sensory input and are of great significance for studying the early sensory processing mechanisms of the auditory system; Endogenous components, including N2, P300, and mismatch negativity (MMN), are closely related to the auditory processing of the higher central nervous system and have received widespread attention and research in the field of neuroscience (X. Li et al. 2023; Benghanem et al. 2022; S. Li et al. 2024).

Due to the significant heterogeneity of tinnitus patients across different studies, as well as differences in the sound stimuli and recording methods used in each study, the research on AERPs in tinnitus patients has not reached a consistent conclusion. Gabr et al. found that tinnitus patients with normal hearing (pure tone hearing threshold< 25 dB HL at various frequencies) exhibit delayed P300 wave latency and decreased amplitude (Gabr et al. 2022). However, some studies have found that there are no significant changes in the amplitude and latency of P300 waves in patients with tinnitus (Houdayer et al. 2015). There have been systematic reviews summarizing the changes in P300 waves in tinnitus patients, and both systematic reviews suggest that tinnitus patients may exhibit significantly reduced P300 amplitude (Cardon et al. 2020; Vasudevan et al. 2021). However, the results regarding P300 latency are not consistent. This further illustrates that the differences in subjects and research methods across various studies have led to biased conclusions between the studies. In addition, although the recording of AERPs is based on cortical activity generated by sound stimuli, severe hearing loss may not apply to this test. However, some studies have found that the hearing level of subjects may affect AERPs (Madashetty et al. 2024; Gommeren et al. 2021). More than 90% of patients with idiopathic tinnitus have varying degrees of hearing loss, and there is currently no research grouping based on hearing level to explore the impact of hearing loss on AERPs in patients with idiopathic tinnitus. However, after integrating primary studies, existing reviews have proposed the hypothesis that hearing loss may affect AERPs in patients with tinnitus, providing a basis for the design of this study's grouping (Jacxsens et al. 2022). This study fills the gap by distinguishing the independent and combined effects of tinnitus itself and mild hearing loss on central auditory processing, which helps solve the heterogeneity problem of previous studies caused by mixed hearing levels.

This study selected the most widely used oddball sound stimulation mode, used standardized AERPs recording methods (Polich and Kok 1995), and adopted strict inclusion and exclusion criteria to collect data from idiopathic tinnitus patients with tinnitus as the chief complaint. Patients with an average hearing threshold > 35 dB HL and significant psychological problems were excluded to minimize the impact of comorbidities. By grouping patients according to hearing level (normal hearing vs. mild hearing loss), this study excludes the interference of severe hearing loss on AERP recording and focuses on mild hearing loss. Through AERPs, we explore the neurophysiological abnormalities of these two subgroups, aiming to reveal: (1) the cognitive dysfunction of idiopathic tinnitus patients, and (2) the specific impact of mild hearing loss on central auditory processing. This design provides a theoretical basis for explaining the mechanism of central plasticity changes and developing central targeted therapy for idiopathic tinnitus.

## 2 Materials and Methods

### 2.1 Subjects

We included 74 patients with idiopathic tinnitus, recruited from the Union Hospital of Tongji Medical College of Huazhong University of Science and Technology. According to the classification criteria for hearing loss in the *2021 World Hearing Report* (Chadha et al. 2021), they were divided into a normal hearing group (TNH, better ear hearing threshold < 20 dB HL) of 39 cases and a mild hearing loss group (THL, better ear hearing threshold 20–35 dB HL) of 35 cases. Meanwhile, we recruited 37 healthy volunteers during the same period as the control group (HC).

To mimic the clinical setting and minimize the impact of comorbidities as much as possible, we adopted the following inclusion criteria: (1) Tinnitus was the primary complaint, and the duration of tinnitus was ≥ 6 months; (2) no underlying cause (excluding sensorineural hearing loss) or identifiable physiological state was found to be associated with tinnitus (i.e., meeting the definition of idiopathic tinnitus) (Tunkel et al. 2014); (3) normal hearing or only mild hearing loss, that is, better ear hearing threshold is less than 35 dB HL. The external auditory canal was unobstructed, and the tympanic membrane was intact; (4) subjects with normal understanding and expression ability. Exclusion criteria: (1) Otitis externa, acute/chronic otitis media, and other related diseases of the external ear and middle ear; (2) sudden deafness, Meniere's disease, otosclerosis, acoustic neuroma, and other inner ear or auditory nerve diseases; (3) severe central nervous system diseases or other systemic diseases such as cardiovascular and cerebrovascular diseases. (4) Any other factors that may affect scalp potential recording, such as maxillofacial scars. Healthy volunteers are individuals who have no tinnitus, no symptoms of anxiety, depression, or sleep disorders, and are physically and mentally healthy with normal hearing.

This study was conducted in accordance with the principles of the Helsinki Declaration. All subjects gave informed consent to a study protocol approved by the local Ethics Committee of Union Hospital of Tongji Medical College of Huazhong University of Science and Technology (Institutional Review Board approval [2024] No. 0196).

### 2.2 Clinical Evaluation

We collected demographic information and audiometry results for all participants. Detailed interviews were conducted to gather medical history and assess the discomfort experienced by tinnitus patients. Each patient with tinnitus underwent evaluations for loudness matching and pitch matching (with Tf indicating the frequency of their tinnitus) using standardized characteristics of the tinnitus sound. In addition, patients completed the Tinnitus Handicap Inventory (THI) to evaluate the severity of their tinnitus.

To evaluate the associated symptoms of tinnitus, we administered the Montreal Cognitive Assessment (MoCA) with permission to assess the cognitive function of all participants. We used the Self‐Rating Anxiety Scale (SAS) and the Self‐Rating Depression Scale (SDS) to assess levels of anxiety and depression, respectively, and employed the Pittsburgh Sleep Quality Index (PSQI) to assess sleep quality.

### 2.3 AERPs Acquisition

#### 2.3.1 Test Protocol

The AERPs test was conducted in a standard auditory electrophysiological shielding room with a background noise of less than 20 dB (A), using the CAEP testing module of the Eclipse (Interacoustics, Middelfart, Denmark).

Adopting standard oddball mode stimulation both in P300 test and MMN test: 1000 Hz tone‐burst as non‐target stimuli in the P300 test and standard stimuli in the MMN test (80%), 2000 Hz tone‐burst as target stimuli in the P300 test and deviant stimuli in the MMN test (20%); these two stimuli are randomly presented by the system according to the set probability. Both types of stimuli have an intensity of 70 dB nHL, rise/fall time = 10 ms, platform duration = 30 ms, stimulation rate of 1.1 times/s, signal averaging ≥ 100 times, and window time = 700 ms.

**FIGURE 1:**
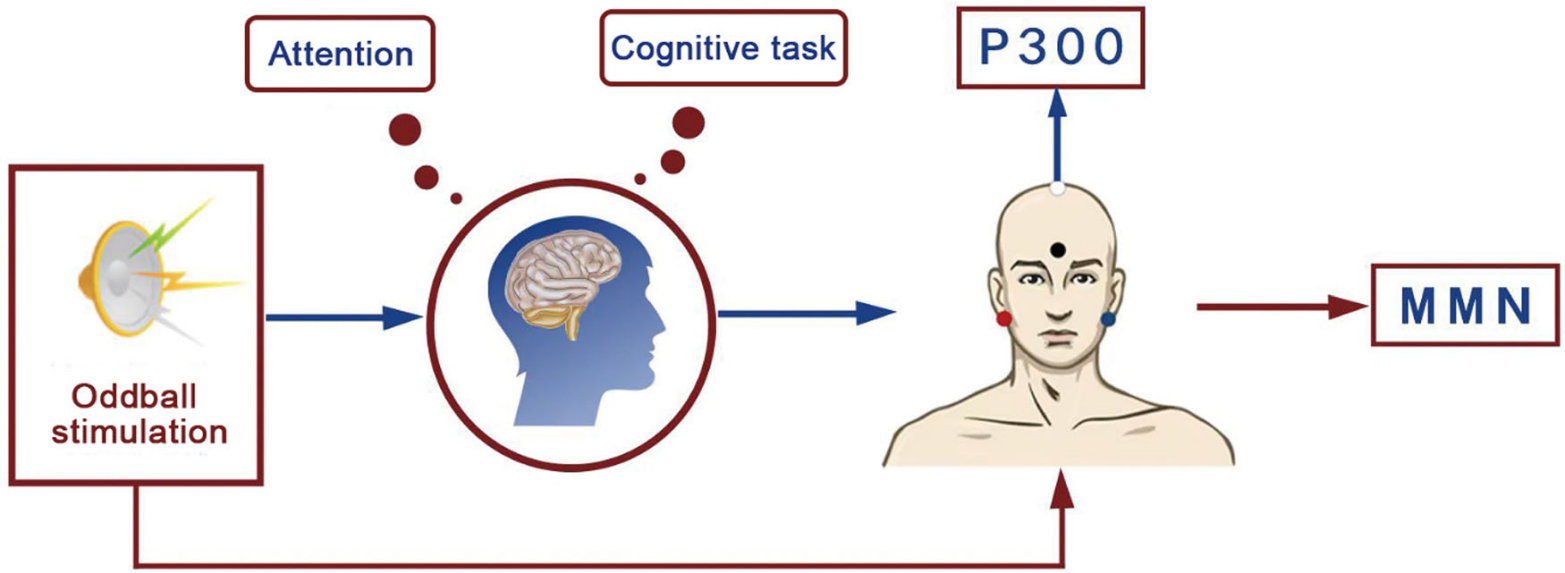
Schematic diagram of AERPs testing. The blue arrow indicates that the P300 testing process requires active attention to stimulus sounds and completion of psychological tasks; the red arrow indicates that MMN testing does not require subjects to pay attention to stimulus sounds; the white electrode is the recording electrode placed in Cz; the black electrode is the grounding electrode placed on Fpz; the red and blue electrodes are reference electrodes placed on M1 and M2.

After skin degreasing, a recording electrode (conductive paste to reduce impedance) is placed at the top of the skull (Cz), the reference electrodes (short‐circuited) are placed at both mastoid processes (M1, M2), and a grounding electrode is placed at the center of the brow (Fpz) (Figure 1). The impedance between each electrode is less than 5 k Ω.

The subjects were instructed to wash their hair before the AERPs test and avoid using hair oil, conditioner, or other hair care products to prevent waveform distortion caused by high scalp resistance. After sufficient rest, the subjects sit comfortably in a standard soundproof room with a background noise level of less than 20 dB (A). They are required to remain awake, concentrate, and avoid blinking, opening their mouth, and swallowing. Ensure that the subjects have a clear understanding of the task and alleviate emotions such as tension and anxiety.

During the optimization of the testing protocol, we discovered that the low background noise (< 20 dB A) and comfortable environment of the soundproof room, combined with the monotonous pure tones generated by the oddball stimulation paradigm, made subjects prone to drowsiness. To address this, subjects were instructed to sit upright and stay awake, with reminders given before each examination to help maintain alertness.

In the MMN testing protocol, subjects were required to remain awake and watch silent videos of their choice to divert their attention while ignoring auditory stimuli. The subjects will undergo P300 testing 30 min after completing the MMN test. Before the P300 test, the subjects were informed that they would hear two types of stimuli and conduct 2–3 pre‐tests to familiarize them with the two stimuli. In contrast to ignoring auditory stimuli in the MMN test, participants are required to remain awake and focus their attention. When they hear a target stimulus sound with a higher pitch and fewer occurrences (2000 Hz), they should press a response button with their thumb quickly and accurately. After testing, it was verified again whether the subjects understood the task and could distinguish between the two types of stimuli based on the accuracy of their responses. Two rounds of testing were performed, with each subject undergoing 2–3 tests on each ear, alternating between left and right ears to ensure reliable results.

Regarding the response recording method used in the P300 test, we found that simply asking subjects to memorize the number of target stimuli could distract them and increase the likelihood of drowsiness. To alleviate this pressure, we employed a counter and excluded waveforms with inaccurate counts from our analysis. Subjects who consistently counted inaccurately or struggled to distinguish between the two types of stimulus sounds after multiple trials were excluded from the study, in accordance with our inclusion and exclusion criteria. These criteria stipulated that participants should have “normal comprehension and expression abilities.”

#### 2.3.2 Waveform Identification

**FIGURE 2:**
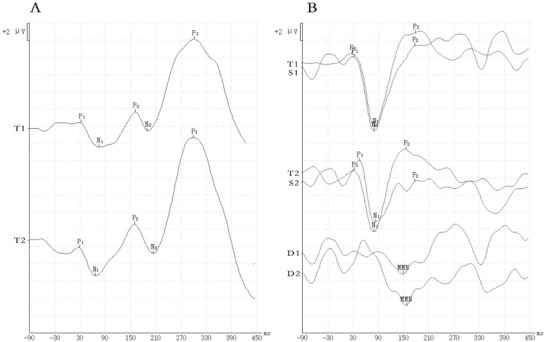
Typical normal subject P300 (A) and MMN (B) waveforms. T: Waveforms induced by target stimuli (deviant stimuli); S: Waveforms induced by non‐target stimuli (standard stimuli); D: the difference waveform obtained by subtracting the two; the number represents the number of tests conducted.

In the P300 test, the waveform induced by the target stimulus (T) consists of a P1 component (first positive wave) with a latency of about 50 ms, an N1 component (first negative wave) with a latency of 80–100 ms, a P2 component (second positive wave) with a latency of 180–200 ms, an N2 component (second negative wave) with a latency of about 250 ms after the P2 component, and a P300 wave (third positive wave) with a latency of about 300 ms (Figure 2A). We recorded the latency and amplitude of P1, N1, P2, N2, and P300. MMN is the maximum difference wave with a latency of 100–250 ms in the difference waveform (D) obtained by subtracting the non‐target stimulus waveform (S) from the target stimulus waveform (T) (Figure 2B), and the latency and amplitude of MMN are recorded. The latency period is the time from 0 ms after the sound is given to the peak of the positive or negative wave. Represent the amplitude as the difference in peak potential between adjacent peaks of opposite polarity. Calculate the mean of two waveforms with good repeatability as the evaluation metric.

**FIGURE 3:**
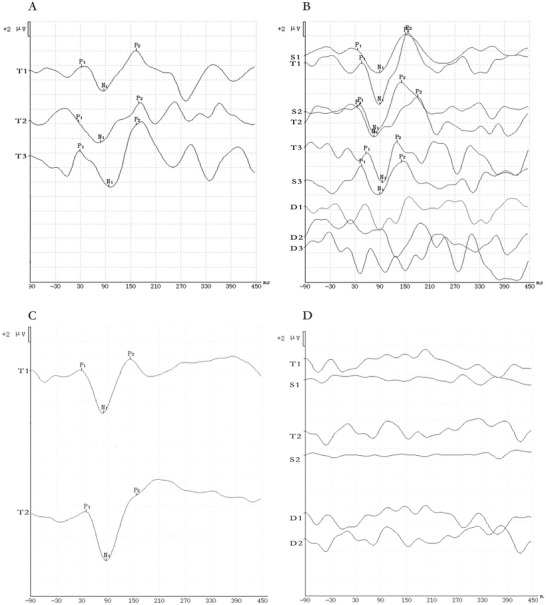
Waveforms without reproducible waves. (A) P300 wave without repetition; (B) MMN wave without repetition; (C) P300 wave was not elicited; (D) MMN wave was not elicited. T: Waveforms induced by target stimuli (deviant stimuli); S: waveforms induced by non‐target stimuli (standard stimuli); D: the difference waveform obtained by subtracting the two; the number represents the number of tests conducted.

However, some patients with idiopathic tinnitus may have difficulty in identifying waveforms for some reason; that is, there is no reproducible wave in the waveform (Figure 3). If the waveform cannot be repeated three times in one ear test, it is considered that the waveform has no repetition (Figure 3A,B); If the corresponding wave cannot be identified (Figure 3C,D), it means that the wave has not been elicited.

This study selected AERPs data from the affected ears of tinnitus patients for analysis, and for patients with bilateral tinnitus, data from the ear with more severe tinnitus were selected. Two audiologists with years of experience in auditory electrophysiology conducted blind evaluations of all waveforms, and waveforms with differing opinions were discussed and determined by the two audiologists. In each test, the latency and amplitude averages of each wave from the two waveforms with the highest repeatability were selected as the final data for statistical analysis.

### 2.4 Statistical Analysis

SPSS 25.0 software (SPSS Inc., Chicago, IL, USA) was utilized for processing clinical data and conducting statistical analyses. The Shapiro‐Wilk test was used to assess the normality of the measurement data, while the Levene test evaluated the homogeneity of variance for the normally distributed data. For measurement data that followed a normal distribution, results were presented as $\bar{x}$± *s*, and a *t*‐test was employed. In contrast, measurement data that did not conform to the normal distribution were summarized as M (P25, P75), and a non‐parametric test was applied. The count data were displayed by frequency, and the chi‐square test was used for analysis.

In the analysis of AERPs, we exclude waveforms that are non‐repetitive and unextracted. Differences in AERPs indicators (latency and amplitude of P1, N1, P2, N2, P300, and MMN) among groups were analyzed using one‐way ANOVA. Include age, gender, education level, SAS score, SDS score, and MoCA score as covariates in the analysis. And conduct correlation analysis between various AERPs indicators and various clinical features.

## 3 Results

### 3.1 Demographic and Clinical Characteristics

As shown in Table 1, there were no significant differences between the HC group and the TNH group in terms of age (*p* = 0.844), gender (*p* = 0.902), education level (*p* = 0.742), and hearing thresholds in the better ear (*p* = 0.889) or worse ear (*p *= 0.644). In terms of subjective scale scores, the MoCA score of the TNH group was significantly lower than that of the HC group (*p *= 0.003). The PSQI score was significantly higher than that of the HC group (p = 0.001), while there were no significant differences in SDS (*p* = 0.458) and SAS (*p* = 0.133) scores.

**TABLE 1:** Demographic and clinical characteristics.

	THL(*n* = 35)	TNH(*n* = 39)	HC(*n* = 37)	Statistics	*p*
Age(years)	53.7 ± 9.6	42.8 ± 13.7	40.7 ± 11.6	*F* = 12.40	< 0.001
Gender(male/female)	17/18	18/21	18/19	*χ*2 = 0.21	0.902
PTA of the better ear	28.3 ± 7.7	13.0 ± 3.9	12.1 ± 7.5	*F* = 70.01	< 0.001
PTA of the worse ear	33.0 ± 9.3	17.5 ± 8.5	15.4 ± 8.2	*F* = 44.39	< 0.001
Education(years)	14.2 (11.9,15.8)	14.6 (12.4,16.0)	14.4 (12.3,16.0)	*H* = 0.60	0.742
MoCA scores	22.9 ± 3.5	24.8 ± 3.6	27.1 ± 1.7	*F* = 17.20	< 0.001
SAS scores	27.6 (22.8,32.3)	27.0 (22.1,32.8)	24.5 (22.0,29.8)	*H* = 1.56	0.458
SDS scores	25.6 (21.9,30.0)	25.4 (22.6,31.8)	23.3 (21.2,26.6)	*H* = 4.04	0.133
PSQI scores	9.7 ± 4.6	8.0 ± 3.8	5.1 ± 2.8	*F* = 13.28	< 0.001
Sides(left/right/bilateral)	9/9/17	10/9/20		*χ*2 = 0.01	0.961
Duration(months)	14.4 (8.6,36.8)	11.6 (7.3,46.0)		*T* = 0.40	0.691
Tf(low/mid/high)	5/9/21	6/9/24		*χ*2 = 0.08	0.963
Tinnitus loudness	58.2 ± 11.7	39.2 ± 17.4		*t* = 5.53	< 0.001
THI scores	26.2 ± 16.3	29.2 ± 16.0		*t* = 0.66	0.512

Abbreviations: HC: healthy control; MoCA: Montreal Cognitive Assessment; PSQI: Pittsburgh Sleep Quality Index; PTA: mean pure tone thresholds at 500, 1000, 2000, and 4000 Hz; SAS: Self‐Rating Anxiety Scale; SDS: Self‐Rating Depression Scale; Tf: tinnitus frequency, low: ≤ 1000 Hz, mid: 1001–4000 Hz, high: > 4000 Hz; THI: Tinnitus Handicap Inventory; THL: idiopathic tinnitus patients with mild hearing loss; TNH: idiopathic tinnitus patients with normal hearing.

Compared with the TNH group and HC group, the THL group showed higher age (*p* < 0.001) and higher hearing thresholds in both better and worse ears (p < 0.001). However, there were no significant differences in gender (*p* = 0.902) and education level (*p* = 0.742).

There was no significant difference in SDS (*p* = 0.458), SAS (*p* = 0.133), and PSQI (*p* = 0.279) scores between the two groups of idiopathic tinnitus patients. Compared with the TNH group, the MoCA score of the THL group showed a decreasing trend (*p* = 0.059), but the difference was not significant. Similarly, there were no significant differences between the TNH group and the THL group in terms of tinnitus disease characteristics, such as tinnitus side (*p* = 0.961), duration of illness (*p* = 0.691), and tinnitus pitch (*p* = 0.963). There was no significant difference in THI (*p* = 0.512) scores between the two groups. THL tinnitus loudness (*p* < 0.001) was significantly higher than TNH.

### 3.2 P300

The elicited rates of P1, N1, P2, N2, and P300 in both ears of 37 subjects in the HC group were all 100%. In the TNH group, five subjects did not show reproducible P300 in their affected ears (5/39, 12.8%); One case of P300 wave was not elicited, and four cases were non‐repetitive. There were also five subjects in the THL group who did not show reproducible P300 waves in their affected ears (5/35, 14.3%). Among them, four cases did not elicit P300, and one case was non‐repetitive. This section only reports the reproducible P300 waveform data.

As shown in Table 2, the latency of N2 (*p* = 0.011) and P300 (*p* < 0.001) in the TNH group was significantly delayed compared to the HC group. The amplitude of P1 (*p* = 0.035) was significantly higher than that of the HC group, while the amplitude of P300 (*p* = 0.031) was significantly lower than that of the HC group. Compared with the HC group, the N2 latency (*p* = 0.006) and P300 latency (*p* = 0.001) of the THL group were significantly delayed, the P1 amplitude (*p* = 0.007) was significantly increased, and the P300 wave amplitude (*p* < 0.001) was significantly decreased. Compared with the TNH group, there was no significant difference in the latency of each wave in the THL group. In terms of amplitude, the amplitude of P300 (*p* = 0.012) decreased significantly, while the amplitude of P2 (*p* = 0.048) increased significantly.

**TABLE 2:** The latency and amplitude of each wave in the P300 test for three groups of subjects.

		THL(*n* = 30)	TNH(*n* = 34)	HC(*n* = 74)	Statistics	*p*
Latency/ms	P1	36.4 ± 11.4	36.2 ± 12.6	35.2 ± 10.2	*F* = 0.17	0.841
	N1	84.0 ± 10.4	86.0 ± 12.1	84.6 ± 14.3	*F* = 0.20	0.820
	P2	163.1 ± 20.9	164.6 ± 26.1	156.2 ± 22.6	*F* = 1.90	0.154
	N2	234.6 ± 29.1	232.2 ± 40.6	214.6 ± 30.1	*F* = 5.63	0.004
	P300	327.8 ± 37.6	332.0 ± 30.1	303.0 ± 37.7	*F* = 10.90	< 0.001
Amplitude/µV	P1	5.6 ± 2.4	5.4 ± 1.4	4.5 ± 1.8	*F* = 4.64	0.011
	N1‐P2	7.8 ± 3.0	6.4 ± 2.3	7.1 ± 3.2	*F* = 1.86	0.160
	P2	5.8 ± 2.7	4.8 ± 2.1	4.4 ± 2.5	*F* = 4.17	0.001
	N2‐P3	6.5 ± 4.6	6.5 ± 3.8	6.3 ± 3.4	*F* = 0.03	0.968
	P3	4.7 ± 2.3	6.7 ± 4.5	8.6 ± 4.6	*F* = 9.94	< 0.001

Abbreviations: HC: healthy control; N2−P3: recording potential of P3−recording potential of N2; NI−P2: recording potential of P2−recording potential of N1; THL: idiopathic tinnitus patients with mild hearing loss; TNH: idiopathic tinnitus patients with normal hearing.

### 3.3 MMN

The elicited rate of MMN waves in both ears of 37 subjects in the HC group was 100%. In the TNH group, seven subjects did not show reproducible MMN waves (7/39, 17.9%), of which three were not elicited, and four were non‐repetitive. In the THL group, 14 subjects did not show reproducible MMN waves (14/35, 40%), of which eight were not elicited, and six were non‐repetitive.

There was no significant difference in MMN extraction rate between the two groups of tinnitus patients (*χ* ^2^ = 4.14, *p* = 0.126). Only the differentiable MMN waveforms are included in the analysis of MMN wave differences.

**FIGURE 4:**
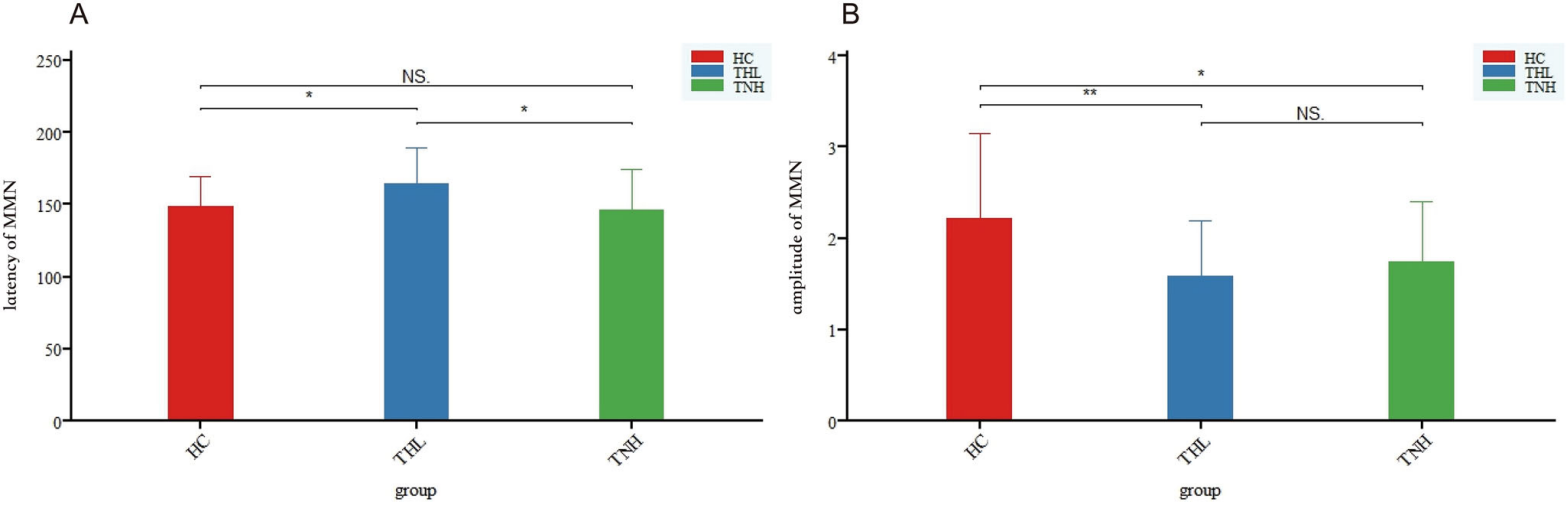
Comparisons of MMN among the three groups. (A) Comparison of MMN latency; (B) comparison of MMN amplitude. HC: healthy control; THL: idiopathic tinnitus patients with mild hearing loss; TNH: idiopathic tinnitus patients with normal hearing; NS: *p* > 0.05; *p < 0.05; **p < 0.01.

The THL group showed a significant delay in MMN latency compared to the TNH group (*p* = 0.011) and HC group (*p* = 0.021), while there was no significant difference in MMN latency between the TNH group and HC group (*p* = 0.717), as shown in Figure 4A.

In terms of MMN amplitude, both the THL group (*p* = 0.004) and TNH group (*p *= 0.014) showed significant reductions compared to the HC group, while there was no significant difference in MMN amplitude between the two groups of tinnitus patients (*p* = 0.473), as shown in Figure 4B.

### 3.4 Correlation Analysis

**FIGURE 5:**
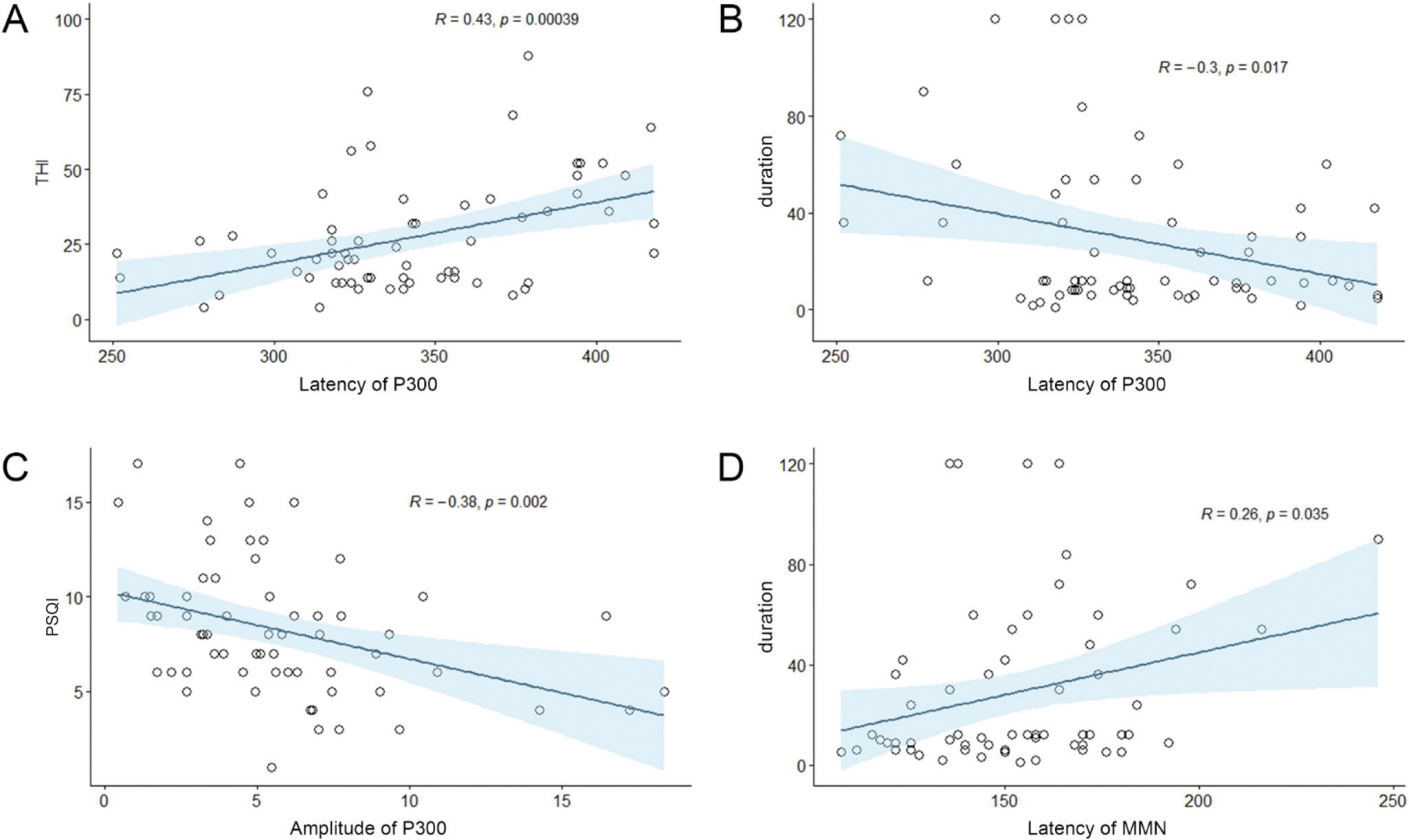
Correlation analysis results between clinical features and AERPs. (A) Scatter plot of P300 latency and THI; (B) scatter plot of P300 latency and duration of tinnitus; (C) scatter plot of P300 amplitude and PSQI; (D) scatter plot of MMN latency and duration of tinnitus.

Select clinical characteristics of tinnitus patients, such as age, gender, duration of illness, THI score, SAS score, SDS score, MOCA score, and PSQI score, and conduct correlation analysis with the neurophysiological indicators of tinnitus patients (latency and amplitude of P1, N1, P2, N2, P300, and MMN). As shown in Figure 5, P300 waves and MMN waves show significant correlation with some clinical features:

1. The latency of P300 is significantly positively correlated with THI score (*R* = 0.43, *p* < 0.001, Figure 5A)
2. The latency of P300 is significantly negatively correlated with the duration of tinnitus (*R* = −0.3, *p* = 0.017, Figure 5B)
3. The amplitude of P300 is significantly negatively correlated with PSQI score (*R* = −0.38, *p* = 0.002, Figure 5C)
4. The latency of MMN is significantly positively correlated with the duration of tinnitus (*R* = 0.26, *p* = 0.035, Figure 5D)

## 4 Discussion

This study aimed to verify abnormal brain electrical activity in patients with idiopathic tinnitus using AERPs and clarify the impact of mild hearing loss. By comparing AERPs indicators between tinnitus patients (grouped by hearing level) and healthy controls, it was found that patients with idiopathic tinnitus had multiple AERPs abnormalities, and mild hearing loss exacerbated some of these abnormalities.

Unlike periodic spontaneous EEG, AERPs are induced by auditory stimuli and have low amplitude, relatively fixed latency, and waveform characteristics. By repeatedly recording and superimposing the evoked EEG waveforms, spontaneous EEG waveforms unrelated to auditory stimuli are mutually canceled out, restoring the electrical activity of auditory‐related neurons induced by the stimulus and reflecting the response characteristics of the central nervous system to auditory stimuli (Habib and Habib 2021; Mcfadden et al. 2021). The characteristics of AERPs include the use of non‐singular auditory stimuli, recording long latency potentials, and assigning special psychological meanings to certain stimuli. These characteristics enable it to reflect the cognitive processing of auditory information by the central nervous system and explore higher‐level central electrophysiological changes (Kamal et al. 2021). These characteristics make it a high‐quality tool for revealing central abnormalities in idiopathic tinnitus.

### 4.1 Lower Elicited Rates of P300 and MMN in Idiopathic Tinnitus

The study revealed that patients with idiopathic tinnitus exhibited greater waveform variability and lower elicited rates compared to the control group, which had a 100% extraction rate and characteristic waveform patterns for P300 and MMN. In previous AERPs studies related to tinnitus, poor waveform repeatability may have been attributed to non‐standard testing procedures and patient status, leading to the exclusion of such waveforms, and no findings regarding variations in waveform elicited rates among tinnitus patients have been reported. However, the repeatability of auditory evoked potentials has been well‐documented in previous studies in other fields, providing a theoretical basis and analytical insights for the present study (Ikeda and Campbell 2024; Kavanagh et al. 1988).

One possible reason for this is that some patients with idiopathic tinnitus may experience central auditory processing disorders due to various factors, hindering the elicitation of P300 and MMN waves. These waves typically show no significant fluctuations at their corresponding latency positions, with amplitudes similar to baseline levels and only minor, unidentifiable artifacts—termed P300/MMN waves not elicited in this study. The other is that the amplitude values of the P300 and MMN waves in idiopathic tinnitus patients were significantly lower than those in the control group. This may have led to confusion with artifacts created by multi‐source electrical activity in the cerebral cortex due to auditory stimulation, resulting in multiple peaks or large plateaus at the corresponding latency. Upon comparing multiple waveform curves, we found a lack of repeatability, which we defined as waveform non‐repeatability.

Regarding the P300 wave analysis, within the group of 39 patients with normal hearing and idiopathic tinnitus, four exhibited a non‐repetitive P300 wave, while one showed that the P300 wave was not elicited. Among the 35 patients with idiopathic tinnitus and mild hearing loss, four did not elicit the P300 wave, and one had no repeatability. This may indicate that the presence of idiopathic tinnitus leads to multiple sources of EEG activity induced by target stimulation, which interferes with the recognition of P300 waves. Furthermore, mild hearing loss may exacerbate central auditory processing disorders, leading to a higher incidence of absent P300 waves. These observations were corroborated in the MMN analysis. Although there was no significant difference in the rate of non‐elicitation of MMN waves, up to 40% of the participants in the THL group did not show reproducible MMN waves. This included eight individuals without elicited MMN waves and six who exhibited non‐repetitive waves. In contrast, in the TNH group, only 17.9% experienced similar outcomes, with three cases showing no elicited MMN waves and four lacking repeatability.

To maintain consistency with the P300 in the stimulus paradigm, reduce the cognitive load on subjects, and enhance the reproducibility of the test paradigm, the present study adopted a relatively large frequency difference between the deviant and standard stimuli in the MMN test (1000 Hz vs. 2000 Hz). This may lead to difficulties in identifying the MMN wave due to the N1‐refractoriness effect (Rosburg et al. 2022), which could be one of the reasons why the reproducibility of the MMN waveform in patients with idiopathic tinnitus is poorer than that of the P300.

However, due to the limited sample size in this study and the lack of relevant waveforms in previous research on tinnitus‐related AERPs, further studies with larger sample sizes are necessary to determine whether abnormalities exist in the waveform elicited rates of AERPs in patients with idiopathic tinnitus.

### 4.2 Abnormal Exogenous Components in Idiopathic Tinnitus

In the exogenous components responsible for the early processing of auditory information, we found that the amplitude of P1 was significantly increased in patients with idiopathic tinnitus. At the same time, there was no significant difference in P1 between the two groups of idiopathic tinnitus patients. Consistent with the results of this study, Konadath and Manjula found that tinnitus patients with normal hearing exhibited a significant increase in P1 amplitude, which was attributed to central gain resulting from ultra‐high‐frequency (> 8 kHz) hearing loss (Konadath and Manjula 2016). After experiencing ultra‐high‐frequency hearing loss, patients with tinnitus often report reduced peripheral input and decreased activity in the auditory cortex. The central nervous system calibrates this imbalance of electrical activity and generates a “tinnitus source” located in the auditory cortex. Dabbous et al. (Dabbous et al. 2024) and Morse and Vander Werff (Morse and Vander Werff 2024) also obtained similar results. In terms of P1 component latency, previous research results have been inconsistent, which may be attributed to differences in the subjects included and the testing methods used (Santos Filha and Matas 2010; Norena et al. 1999). In this study, no statistically significant differences in P1 latency were found.

Gopal et al. found that patients with bilateral chronic tinnitus may have an increased amplitude of N1 components. Based on fMRI, the increased amplitude N1 components in tinnitus patients were located in specific brain regions, which may exhibit excessive activity during high‐intensity pure tone stimulation near their tinnitus frequency (Gopal et al. 2017). Gopal et al. explained that tinnitus patients may have a selective attention deficit, where these brain regions are allocated excessive attention, leading to chronic and habituation disorders in tinnitus (Gopal et al. 2017). Although the subjects included in this study also had chronic tinnitus (lasting more than 6 months), there was no significant difference in the amplitude of the N1 component in the results of this study. This may be due to inconsistent calculation methods for waveform amplitude. In this study, the amplitude of the negative wave, that is, N1‐P2 or N2‐P3, was calculated by calculating the absolute difference between the peak of the positive wave and the subsequent positive wave.

However, this study found a significant increase in P2 amplitude in idiopathic tinnitus patients with mild hearing loss. At the same time, there was no significant difference between patients with TNH and the control group. This finding is consistent with previous research, further validating the central gain hypothesis that patients with mild hearing loss are more likely to exhibit abnormalities in the exogenous component of cortical evoked potentials due to a more significant reduction in peripheral input (Ralston et al. 2024; Morse and Vander Werff 2023).

### 4.3 P300 abnormality Indicates Selective Attention Disorder in Idiopathic Tinnitus

The P300 wave can comprehensively reflect the subject's selective attention, appearing only when the subject actively pays attention to target stimuli with a lower frequency and responds after receiving the task, reflecting top‐down and bottom‐up attention.

This study found that patients with idiopathic tinnitus exhibited significant changes in the P300 wave, including delayed latency and decreased amplitude. This is consistent with most previous research findings (Gabr et al. 2022; Cardon et al. 2020; Vasudevan et al. 2022; Alonso‐Valerdi et al. 2023). Asadpour et al. recorded the P300 waves of tinnitus patients with normal hearing using 32‐channel electroencephalography and found that the P300 amplitude measured by multi‐channel was significantly reduced (Asadpour et al. 2018), which corroborates the results of the present study. A few studies have reported that the changes in P300 in tinnitus patients may not be significant, which can be attributed to the fact that the included subjects may not represent the true characteristics of most tinnitus patients (Houdayer et al. 2015).

Zhao et al. found that patients with age‐related hearing loss exhibit delayed P300 latency and decreased amplitude, suggesting that hearing loss may also cause attention deficit and abnormal P300 waves (Zhao et al. 2024). However, as the most common comorbid symptom of tinnitus, previous studies have not explored the effect of hearing loss on P300 in patients with idiopathic tinnitus. This study found that mild hearing loss does not affect the latency of the P300 in patients with idiopathic tinnitus, but it further reduces the amplitude of the P300 in these patients. This suggests that mild hearing loss does not significantly prolong the central auditory processing time during the attention process in patients with idiopathic tinnitus, but leads to a reduction in the number of neurons involved in the attention process or a decrease in discharge synchrony.

In addition, this study also found that some clinical features of patients with idiopathic tinnitus were significantly correlated with P300 waves. A significant positive correlation exists between P300 latency and THI score in patients with idiopathic tinnitus, suggesting that those with severe tinnitus interference are more likely to exhibit abnormal P300 latencies. This finding is highly consistent with the research results of Wang et al., which suggest that patients with severe tinnitus are more prone to selective attention deficit (Näätänen et al. 2012). Moreover, this study also found a significant negative correlation between the duration of tinnitus and P300 latency, which seems unreasonable and counterintuitive. Previous studies have not examined the relationship between tinnitus duration and changes in P300 latency. This study speculates that this may be related to the better adaptation of patients with a longer duration of idiopathic tinnitus to tinnitus and lower THI scores. Further research is needed to include patients with different durations of idiopathic tinnitus for in‐depth discussion and exploration. This study also found a significant negative correlation between P300 amplitude and PSQI score in patients with idiopathic tinnitus, suggesting that sleep disorders may be a factor leading to selective attention deficit in patients with idiopathic tinnitus.

### 4.4 MMN Abnormality Indicates a Preprocessing Dysfunction in Idiopathic Tinnitus

MMN reflects the preprocessing of auditory information in the brain, which is generally believed to originate from the bilateral auditory cortex and be regulated by the right frontal lobe (Näätänen et al. 2012).

Although the sample size for MMN analysis was relatively small (*n* > 20) after excluding subjects with non‐reproducible MMN components, the present study still obtained meaningful results that are consistent with previous findings. Wang et al. (Wang et al. 2022) and Sendesen et al. (Sendesen et al. 2022) reported that tinnitus patients have significantly reduced MMN amplitude, which is consistent with the present findings.

This study found that patients with idiopathic tinnitus had significantly reduced MMN amplitude compared to the control group. At the same time, there was no statistically significant difference in MMN amplitude between the two groups of idiopathic tinnitus patients. Mahmoudian et al. adopted the multi‐feature MMN paradigm and found that the MMN amplitude and area under the curve in tinnitus patients were significantly reduced in response to frequency, duration, and silent gap deviant stimuli (Mahmoudian et al. 2013), which is consistent with the results of the present study. This suggests that patients with idiopathic tinnitus may have auditory information preprocessing dysfunction, characterized by reduced neural synchrony in both the auditory cortex and the right frontal lobe. In contrast, mild hearing loss does not exacerbate this dysfunction further. In addition, the present study found that THL patients had significantly longer MMN latency than both TNH patients and HC subjects. This suggests that mild hearing loss is associated with MMN abnormalities, and tinnitus may not be a direct factor affecting MMN latency, which is consistent with the findings of Chen et al. (Chen et al. 2022). In addition, contrary to the P300 latency, the MMN latency is significantly and positively correlated with the duration of tinnitus in patients, indicating that the chronicity of tinnitus plays a crucial role in the preprocessing dysfunction of auditory information. It is worth noting that Mahmoudian et al. induced residual inhibition (RI) and found that the MMN amplitude and area under the curve in tinnitus patients of the RI group were significantly increased (Mahmoudian et al. 2015). This confirms that the selective attention deficit in tinnitus patients can be improved through intervention, providing key evidence for the central mechanism of tinnitus‐related attention deficits.

## 5 Strengths and Limitations

This study collected AERPs from patients with idiopathic tinnitus and analyzed their neurophysiological characteristics based on AERPs to evaluate the central auditory processing disorders associated with idiopathic tinnitus. In addition, Vasudevan et al. emphasized that hearing loss is a strong confounding factor in evaluating cognitive impairment in tinnitus patients through AERPs, which may lead to biased results (Vasudevan et al. 2021). Although some studies have reported the inclusion of hearing levels in patients with tinnitus, no research has discussed the effects of grouping patients based on hearing levels on AERPs in those with idiopathic tinnitus (Cardon et al. 2022). This study fills this gap by restricting the study to patients with tinnitus as their primary complaint, using strict inclusion and exclusion criteria, which further reduces the heterogeneity of the included subjects. In addition, to improve its reference ability, this study adopted standard AERPs testing methods and conducted detailed waveform analysis and discussion, to standardize future research on AERPs in tinnitus.

However, this study also has some limitations. Firstly, as a cross‐sectional observational study, it did not compare neurophysiological data before and after intervention, and lacks analysis and discussion on how tinnitus progression and outcomes affect AERPs. Secondly, certain data were excluded during waveform analysis, which reduced the statistical power of some analyses. The excluded patients may have more severe central auditory processing disorders or abnormal neuronal synchrony, and this phenomenon itself may be regarded as a potential indicator of central abnormalities in idiopathic tinnitus, providing a new direction for future research. Future studies should expand the sample size; in addition, incorporating techniques capable of localizing relevant brain regions, such as functional magnetic resonance imaging and whole‐brain multi‐channel electroencephalography, will be more helpful for elucidating the central mechanism of tinnitus.

## 6 Conclusion

In summary, patients with idiopathic tinnitus exhibited abnormalities in multiple AERPs indices, and mild hearing loss may exacerbate these abnormalities in some indices. This suggests that patients with idiopathic tinnitus may exhibit central gain secondary to reduced peripheral input, along with selective attention and preprocessing dysfunction. Patients with severe tinnitus may exhibit more pronounced AERPs abnormalities, and the impact of the chronicity of tinnitus on AERPs still requires further research to clarify.

## Author Contributions

Study concept and design: Bo Liu and Hongjun Xiao. Acquisition of data: Xingqian Shen, Hui Pan, Wen Xie, and Linlin Wang. Analysis and interpretation of data: Xingqian Shen and Yingzhao Liu. Drafting of the manuscript: Xingqian Shen and Hui Pan. Critical revision of the manuscript for important intellectual content: Ziying Xu, Xiaoye Chen, Qin Liu, Kaijun Xia. All authors read and approved the final manuscript. Furthermore, all authors are willing to allow the journal to review their data if requested. All authors meet the criteria for authorship stated in the Uniform Requirements for Manuscripts Submitted to Biomedical Journals.

## Funding

This work was supported by the National Key Research and Development Program of China (Nos. 2023YFC2508400, 2023YFC2508000, and 2023YFC2508001), the National Natural Science Foundation of China (NSFC Nos. 81670930, 82101231), and the Natural Science Foundation of Hubei Province, China (No. 2021CFB547).

## Conflicts of Interest

The authors declare no conflicts of interest.

## Data Availability

Data will be made available upon reasonable request.

## References

[brb371308-bib-0001] De Ridder, D. , W. Schlee , S. Vanneste , et al. 2021. “Tinnitus and Tinnitus Disorder: Theoretical and Operational Definitions (An International Multidisciplinary Proposal).” Progress in Brain Research 260: 1–25. 33637213 10.1016/bs.pbr.2020.12.002

[brb371308-bib-0002] Tunkel, D. E. , C. A. Bauer , G. H. Sun , et al. 2014. “Clinical Practice Guideline: Tinnitus Supplement.” Otolaryngology–Head and Neck Surgery 151, no. S2: S1–S40. 25273878 10.1177/0194599814545325

[brb371308-bib-0003] Shore, S. E. , L. E. Roberts , and B. Langguth . 2016. “Maladaptive Plasticity in Tinnitus—Triggers, Mechanisms and Treatment.” Nature Reviews Neurology 12, no. 3: 150–160. 10.1038/nrneurol.2016.12. 26868680 PMC4895692

[brb371308-bib-0004] Baguley, D. , D. Mcferran , and D. Hall . 2013. “Tinnitus.” Lancet 382, no. 9904: 1600–1607. 10.1016/S0140-6736(13)60142-7. 23827090

[brb371308-bib-0005] Li, X. , W. Deng , R. Xue , et al. 2023. “Auditory Event‐Related Potentials, Neurocognition, and Global Functioning in Drug Naïve First‐Episode Schizophrenia and Bipolar Disorder.” Psychological Medicine 53, no. 3: 785–794. 10.1017/S0033291721002130. 34474699

[brb371308-bib-0006] Benghanem, S. , E. Pruvost‐Robieux , E. Bouchereau , M. Gavaret , and A. Cariou . 2022. “Prognostication After Cardiac Arrest: How EEG and Evoked Potentials May Improve the Challenge.” Annals of Intensive Care 12, no. 1: 111. 10.1186/s13613-022-01083-9. 36480063 PMC9732180

[brb371308-bib-0007] Li, S. , C. Lu , N. Liu , et al. 2024. “Association Between Auditory P300 Event‐Related Potential and Suicidal Thoughts and Behaviors in First‐Episode Antipsychotic‐Naïve Patients With Schizophrenia.” Schizophrenia Research 274: 352–359. 10.1016/j.schres.2024.10.015. 39490216

[brb371308-bib-0008] Gabr, T. A. , H. F. Alshabory , and M. A. Kotait . 2022. “Tinnitus: Impact on Patients in Relation to Audiological Findings.” Journal of Laryngology and Otology 136, no. 8: 760–764. 10.1017/S002221512100459X. 35152930

[brb371308-bib-0009] Houdayer, E. , R. Teggi , S. Velikova , et al. 2015. “Involvement of Cortico‐Subcortical Circuits in Normoacousic Chronic Tinnitus: A Source Localization EEG Study.” Clinical Neurophysiology 126, no. 12: 2356–2365. 10.1016/j.clinph.2015.01.027. 25753907

[brb371308-bib-0010] Cardon, E. , I. Joossen , H. Vermeersch , et al. 2020. “Systematic Review and Meta‐Analysis of Late Auditory Evoked Potentials as a Candidate Biomarker in the Assessment of Tinnitus.” PLoS ONE 15, no. 12: e0243785. 10.1371/journal.pone.0243785. 33332414 PMC7746183

[brb371308-bib-0011] Vasudevan, H. , K. Ganapathy , H. P. Palaniswamy , G. Searchfield , and B. Rajashekhar . 2021. “Systematic Review and Meta‐Analysis on the Effect of Continuous Subjective Tinnitus on Attention and Habituation.” PeerJ 9: e12340. 10.7717/peerj.12340. 34900408 PMC8628620

[brb371308-bib-0012] Madashetty, S. , H. P. Palaniswamy , and B. Rajashekhar . 2024. “Investigating the Impact of Hearing Loss on Attentional Networks Among Older Individuals: An Event‐Related Potential Study.” Cognitive Neurodynamics 18, no. 5: 3093–3105. 10.1007/s11571-024-10140-x. 39555299 PMC11564462

[brb371308-bib-0013] Gommeren, H. , J. Bosmans , E. Cardon , et al. 2021. “Cortical Auditory Evoked Potentials in Cognitive Impairment and Their Relevance to Hearing Loss: A Systematic Review Highlighting the Evidence Gap.” Frontiers in Neuroscience 15: 781322. 10.3389/fnins.2021.781322. 34867176 PMC8637533

[brb371308-bib-0014] Jacxsens, L. , J. De Pauw , E. Cardon , et al. 2022. “Brainstem Evoked Auditory Potentials in Tinnitus: A Best‐Evidence Synthesis and Meta‐Analysis.” Frontiers in Neurology 13: 941876. 10.3389/fneur.2022.941876. 36071905 PMC9441610

[brb371308-bib-0015] Polich, J. , and A. Kok . 1995. “Cognitive and Biological Determinants of P300: An Integrative Review.” Biological Psychology 41, no. 2: 103–146. 10.1016/0301-0511(95)05130-9. 8534788

[brb371308-bib-0016] Chadha, S. , K. Kamenov , and A. Cieza . 2021. “The World Report on Hearing, 2021.” Bulletin of the World Health Organization 99, no. 4: 242–242A. 10.2471/BLT.21.285643. 33953438 PMC8085630

[brb371308-bib-0017] Habib, S. H. , and S. S. Habib . 2021. “Auditory Brainstem Response: An Overview of Neurophysiological Implications and Clinical Applications ‐A Narrative Review.” JPMA The Journal of the Pakistan Medical Association 71, no. 9: 2230–2236. 34580520 10.47391/JPMA.03-432

[brb371308-bib-0018] Mcfadden, D. , C. A. Champlin , M. H. Pho , E. G. Pasanen , M. M. Maloney , and E. M. Leshikar . 2021. “Auditory Evoked Potentials: Differences by Sex, Race, and Menstrual Cycle and Correlations With Common Psychoacoustical Tasks.” PLoS ONE 16, no. 5: e0251363. 10.1371/journal.pone.0251363. 33979393 PMC8115856

[brb371308-bib-0019] Kamal, F. , C. Morrison , K. Campbell , and V. Taler . 2021. “Event‐Related Potential Evidence That Very Slowly Presented Auditory Stimuli Are Passively Processed Differently in Younger and Older Adults.” Neurobiology of Aging 103: 12–21. 10.1016/j.neurobiolaging.2021.02.014. 33774574

[brb371308-bib-0020] Ikeda, K. , and T. A. Campbell . 2024. “Binaural Interaction in Human Auditory Brainstem and Middle‐Latency Responses Affected by Sound Frequency Band, Lateralization Predictability, and Attended Modality.” Hearing Research 452: 109089. 10.1016/j.heares.2024.109089. 39137721

[brb371308-bib-0021] Kavanagh, K. T. , P. L. Crews , W. D. Domico , and V. A. Mccormick . 1988. “Comparison of the Intrasubject Repeatability of Auditory Brain Stem and Middle Latency Responses Elicited in Young Children.” Annals of Otology, Rhinology and Laryngology 97, no. 3 pt. 1: 264–271. 10.1177/000348948809700310. 3377393

[brb371308-bib-0022] Rosburg, T. , M. Weigl , and R. Mager . 2022. “No Evidence for Auditory N1 Dishabituation in Healthy Adults After Presentation of Rare Novel Distractors.” International Journal of Psychophysiology 174: 1–8. 10.1016/j.ijpsycho.2022.01.013. 35104580

[brb371308-bib-0023] Konadath, S. , and P. Manjula . 2016. “Auditory Brainstem Response and Late Latency Response in Individuals With Tinnitus Having Normal Hearing.” Intractable & Rare Diseases Research 5, no. 4: 262–268. 10.5582/irdr.2016.01053. 27904821 PMC5116861

[brb371308-bib-0024] Dabbous, A. O. , N. Ali Hosni , A. Al‐Sayed Owais Emara , and A. Said El‐Antably . 2024. “P1‐N1‐P2 Cortical Auditory Evoked Potentials in Chronic Unilateral Acquired Conductive Hearing Loss in Adults.” Journal of International Advanced Otology 20, no. 3: 216–224. 10.5152/iao.2024.231270. 39128038 PMC11232054

[brb371308-bib-0025] Morse, K. , and K. R. Vander Werff . 2024. “Cortical Auditory Evoked Potential Indices of Impaired Sensory Gating in People With Chronic Tinnitus.” Ear and Hearing 45, no. 3: 730–741. 10.1097/AUD.0000000000001463. 38273451

[brb371308-bib-0026] Santos Filha, V. A. V. D. , and C. G. Matas . 2010. “Late Auditory Evoked Potentials in Individuals With Tinnitus.” Brazilian Journal of Otorhinolaryngology 76, no. 2: 263–270. 20549090 10.1590/S1808-86942010000200019 PMC9446206

[brb371308-bib-0027] Norena, A. , H. Cransac , and S. Chéry‐Croze . 1999. “Towards an Objectification by Classification of Tinnitus.” Clinical Neurophysiology: Official Journal of the International Federation of Clinical Neurophysiology 110, no. 4: 666–675. 10.1016/S1388-2457(98)00034-0. 10378736

[brb371308-bib-0028] Gopal, K. V. , B. P. Thomas , R. Nandy , D. Mao , and H. Lu . 2017. “Potential Audiological and MRI Markers of Tinnitus.” Journal of the American Academy of Audiology 28, no. 8: 742–757. 10.3766/jaaa.16106. 28906245

[brb371308-bib-0029] Ralston, L. , J. Campbell , P. Gilley , M. Nielson , and K. Brown . 2024. “Sensory Gating Networks in Normal‐Hearing Adults With Minimal Tinnitus.” American Journal of Audiology 33: 292–302. 10.1044/2023_AJA-23-00122. 38241669

[brb371308-bib-0030] Morse, K. , and K. R. Vander Werff . 2023. “Onset‐Offset Cortical Auditory Evoked Potential Amplitude Differences Indicate Auditory Cortical Hyperactivity and Reduced Inhibition in People With Tinnitus.” Clinical Neurophysiology: Official Journal of the International Federation of Clinical Neurophysiology 149: 223–233. 10.1016/j.clinph.2023.02.164. 36963993

[brb371308-bib-0031] Vasudevan, H. , H. P. Palaniswamy , R. Balakrishnan , et al. 2022. “Cortical Reorganization Following Psychoeducational Counselling and Residual Inhibition Therapy (RIT) in Individuals With Tinnitus.” International Archives of Otorhinolaryngology 26, no. 4: e701–e711. 36405488 10.1055/s-0042-1743287 PMC9668419

[brb371308-bib-0032] Alonso‐Valerdi, L. M. , D. I. Ibarra‐Zárate , A. S. Torres‐Torres , D. M. Zolezzi , N. E. Naal‐Ruiz , and J. Argüello‐García . 2023. “Comparative Analysis of Acoustic Therapies for Tinnitus Treatment Based on Auditory Event‐Related Potentials.” Frontiers in Neuroscience 17: 1059096. 10.3389/fnins.2023.1059096. 37081936 PMC10111057

[brb371308-bib-0033] Asadpour, A. , A. Alavi , M. Jahed , and S. Mahmoudian . 2018. “Cognitive Memory Comparison Between Tinnitus and Normal Cases Using Event‐Related Potentials.” Frontiers in Integrative Neuroscience 12: 48. 10.3389/fnint.2018.00048. 30369872 PMC6194311

[brb371308-bib-0034] Zhao, R. , T. Yue , Z. Xu , et al. 2024. “Electroencephalogram‐Based Objective Assessment of Cognitive Function Level Associated With Age‐Related Hearing Loss.” Geroscience 46, no. 1: 431–446. 10.1007/s11357-023-00847-w. 37273160 PMC10828275

[brb371308-bib-0035] Näätänen, R. , T. Kujala , C. Escera , et al. 2012. “The Mismatch Negativity (MMN)–A Unique Window to Disturbed Central Auditory Processing in Ageing and Different Clinical Conditions.” Clinical Neurophysiology: Official Journal of the International Federation of Clinical Neurophysiology 123, no. 3: 424–458. 10.1016/j.clinph.2011.09.020. 22169062

[brb371308-bib-0036] Wang, K. , X. Lu , and S. Sun . 2022. “Application of Auditory Mismatch Negativity in Tinnitus Patients Based on High‐Resolution Electroencephalogram Signals.” Translational Neuroscience 13, no. 1: 460–469. 10.1515/tnsci-2022-0264. 36561287 PMC9743199

[brb371308-bib-0037] Sendesen, E. , N. Erbil , and M. D. Türkyılmaz . 2022. “The Mismatch Negativity Responses of Individuals With Tinnitus With Normal Extended High‐Frequency Hearing‐Is It Possible to Use Mismatch Negativity in the Evaluation of Tinnitus?.” European Archives of Oto‐Rhino‐Laryngology 279, no. 7: 3425–3434. 10.1007/s00405-021-07097-6. 34564749

[brb371308-bib-0038] Mahmoudian, S. , M. Farhadi , M. Najafi‐Koopaie , et al. 2013. “Central Auditory Processing During Chronic Tinnitus as Indexed by Topographical Maps of the Mismatch Negativity Obtained With the Multi‐Feature Paradigm.” Brain Research 1527: 161–173. 10.1016/j.brainres.2013.06.019. 23810454

[brb371308-bib-0039] Chen, J. , Y. Zhao , T. Zou , et al. 2022. “Sensorineural Hearing Loss Affects Functional Connectivity of the Auditory Cortex, Parahippocampal Gyrus and Inferior Prefrontal Gyrus in Tinnitus Patients.” Frontiers in Neuroscience 16: 816712. 10.3389/fnins.2022.816712. 35431781 PMC9011051

[brb371308-bib-0040] Mahmoudian, S. , M. Farhadi , M. Mohebbi , et al. 2015. “Alterations in Auditory Change Detection Associated With Tinnitus Residual Inhibition Induced by Auditory Electrical Stimulation.” Journal of the American Academy of Audiology 26, no. 4: 408–422. 10.3766/jaaa.26.4.8. 25879244

[brb371308-bib-0041] Cardon, E. , H. Vermeersch , I. Joossen , et al. 2022. “Cortical Auditory Evoked Potentials, Brain Signal Variability and Cognition as Biomarkers to Detect the Presence of Chronic Tinnitus.” Hearing Research 420: 108489. 10.1016/j.heares.2022.108489. 35354098

